# Serum hemato-immunological biomarkers associated with increased COVID-19 mortality in the Latino population

**DOI:** 10.2217/bmm-2022-0056

**Published:** 2022-05-16

**Authors:** Carla PS Ríos, Oscar GJ Cabrera, Juan JJ-V Whaley, Marisol D Sosa, Victor HA Palacios, Guadalupe LH González, José CJ López

**Affiliations:** ^1^Department of Pulmonary Medicine, Instituto Nacional de Enfermedades Respiratorias (INER), Mexico City, ZP, 14080, Mexico; ^2^Department of Pulmonary Medicine, Centro Médico Instituto de Seguridad Social del Estado de México y Municipios (ISSEMyM), Lic. Arturo Montiel Rojas, Metepec, State of Mexico, ZP, 52170, Mexico; ^3^Health Science Research Center (CICSA), Universidad Anáhuac México, State of Mexico, ZP, 52786, Mexico; ^4^Department of Intensive Obstetrics Care Unit, Instituto Materno Infantil, Toluca, State of Mexico, ZP, 50170, Mexico; ^5^Department of Internal Medicine, Centro Médico Instituto de Seguridad Social del Estado de México y Municipios (ISSEMyM), Lic. Arturo Montiel Rojas, Metepec, State of Mexico, ZP, 52170, Mexico; ^6^Department of Thoracic Oncology, Instituto Nacional de Enfermedades Respiratorias (INER) Mexico City, ZP, 14080, Mexico; ^7^Department of Earth Sciences, Universidad Nacional Autónoma de México, Mexico City, ZP, 04510, Mexico

**Keywords:** COVID-19, inflammatory biomarkers, mortality, prognostic factors

## Abstract

**Introduction:** COVID-19 hospitalizations and deaths have raised the need of identifying prognostic factors for medical decision-making. **Methods:** Observational, retrospective study analyzing 191 COVID-19 patients' serum inflammatory biomarkers. **Results:** The median age was 48.7 ± 12.7 years, 75.9% being men. Overweight/obesity was the most common comorbidity in 83.8% of patients. 44.5% had moderate disease followed by 43.5% with severe disease. The mean percentage of pulmonary damage was 53.4% ± 28.7. Serum leukocyte-to-lymphocyte ratio >7.7, neutrophil-to-lymphocyte ratio >10, platelet-to-lymphocyte ratio ≥250 and nutritional index <362 all were independent mortality predictors for COVID-19. **Conclusions:** A leukocyte-to-lymphocyte ratio >7.7 as well as a nutritional index <362 at hospitalization were independently associated with an increased mortality.

## Tweetable abstract

Specific serological inflammatory biomarkers including leukocyte-to-lymphocyte ratio >7.7 and nutritional index <362 are important mortality predictor factors that must be used in the management of COVID-19 hospitalized patients.

In December 2019, the first case of a SARS known as coronavirus pneumonia was diagnosed in Wuhan, China, and in a few months, it was considered a pandemic [1–4]. The disease caused by SARS-CoV-2, which WHO named COVID-19, produces symptoms such as fever, dry cough, dyspnea, fatigue, lymphopenia and anosmia. In severe cases, it leads to a viral pneumonia, SARS and even death [5–7].

As COVID-19 spread, the number of deaths increased rapidly due to limited knowledge of the disease, lack of effective treatments, insufficient health resources and elevated rates of hospital admissions. As knowledge of its pathophysiology increased, organizations and guidelines helped fight the pandemic via facemasks, hand washing, revolutionary vaccines and pharmacological treatments including steroids and immunomodulators. In general, therapy consists of supportive treatment, the use of antiviral therapy and systemic corticosteroids in individualized cases [8]. The WHO COVID-19 panel revealed that the impact on morbidity and mortality was greater than expected, raising awareness in identifying prognostic factors to be used in decision-making by medical personnel.

Inflammation is led by the infectious agent SARS-CoV-2, causing a viral pneumonia and leading to a severe inflammatory response contributing to a weak adaptive immunity and an imbalanced immune response. Due to the severe inflammation, circulating inflammatory biomarkers work as potential prognosis predictor factors [9]. Known indicators of systemic inflammation include total circulating leukocytes, the leukocyte-to-lymphocyte ratio (LLR), neutrophils-to-lymphocyte ratio (NLR), derived neutrophil-to-lymphocyte ratio (d-NLR), platelet-to-lymphocyte ratio (PLR) and monocyte-to-lymphocyte ratio (MLR), which have been studied in patients with inflammatory states including viral pneumonias [10–12].

The NLR has been studied as a prognostic factor in autoimmune diseases, bacterial pneumonia, tuberculosis and neoplasms [13–18]. Nevertheless, the evidence of the NLR as a prognostic factor in COVID-19 remains uncertain. Yuwei Liu *et al.* demonstrated that a higher NLR was associated with a worse outcome in critical patients and was an independent risk factor to predict in-hospital mortality [1]. A different study, by Yang *et al.*, evaluated different ratios including the NLR, d-NLR, PLR and MLR, demonstrating that a threshold ≥3.3 for the NLR helped as a prognostic factor and was associated with a change from mild to severe disease and that patients with an NLR ≤3.3 were most likely to be cured without complications [19]. Jingyuan Liu *et al.* stratified the NLR according to age and demonstrated that patients <50 years with a ratio <3.13 were unlikely to develop severe disease and did not need hospital treatment, but patients >50 years with a ratio ≥3.13 were at higher risk of severe disease and needed hospital care [12].

Due to the promising results of the NLR, d-NLR, PLR and MLR as prognostic factors in systemic inflammatory diseases, including COVID-19, the goal of the current research was to evaluate the impact that these prognostic factors had in disease mortality in the Mexican/Latino population. Most of the studies analyzing these hematological and immunological ratios have been studied in the Asian population and only few studies have established the impact of these as prognostic factors in the Latino population [20]. This study analyzes these ratios in the Latino population, which is extremely important due to the variability of presentation and outcome that COVID-19 has.

In Mexico during the pandemic, the Instituto Nacional de Enfermedades Respiratorias (INER) and the Centro Médico, Instituto de Seguridad Social del Estado de México y Municipios (ISSEMyM) were considered as centers of national reference in the management of COVID-19 patients. Due to the large number of cases diagnosed daily in the respiratory triage area, it is necessary to identify inflammatory biomarkers that are feasible and inexpensive to measure, allowing the identification of patients with a higher risk of severe disease and mortality. These prognostic factors would help with decision-making in COVID-19 care centers, allowing them to prioritize treatment, manage resources and avoid hospital oversaturation.

## Methods

The present study is an observational, retrospective, cross-sectional study that included patients diagnosed with COVID-19 in the INER in Mexico City and the ISSEMyM in Toluca. Patients were analyzed between 13 March and 25 June 2020, which corresponded with the first reported cases of COVID-19 in Mexico. The analysis included sociodemographic variables, comorbidities, clinical symptoms, clinical parameters using the pneumonia severity scale proposed by INER and outcome. Inferential statistics were used considering a significant p-value <0.05, with chi-square tests for nominal and ordinal variables, and variance analysis. Depending on paired or unpaired conditions, the Welch *t*-test, Wilcoxon signed-rank test, or the Mann–Whitney *U* test were used, and the Kruskal–Wallis test used in variables with an interval scale. The full statistical analysis was performed using R software (version 3.6.3). To analyze the relationships between cellularity indices and biochemical markers including the LLR, NLR, PLR and nutritional index (NI) calculated by 10 × serum albumin [g/dl] + (0.005 × lymphocytes/μl), blood samples were obtained at hospital admission. Blood samples were obtained at the moment of hospital admission and analyzed with the following tubes and equipment for their respective blood analysis:

- Yellow tube (blood chemistry and D-dimer) from BD Vacutainer, which contains spray-coated silica and a polymer gel for serum separation. The samples spent 30 min in the tube before analysis and are centrifuged by the Chemistry Analyzer from Beckman Coulter model DxC700AU series number 2018040345 at a speed of 3500 r.p.m for 10 min with a relative centrifugal force of 1000–1300;
- Blue tube (coagulation studies) from BD Vacutainer, which contains buffered sodium citrate solutions 0.109 M, tube size 13 × 75 mm (2.7 ml). The samples spent 30 min in the tube before analysis and are centrifuged with the equipment STA Compact Max from Stago/Licon series number C648034416 at 20 °C with a speed of 2500 r.p.m for 15 min and a relative centrifugal force of 2000–2500;
- Lilac tube (whole blood hematology) from BD Vacutainer, which contains spray-coated K2EDTA, tube size 13 × 75 mm (4.0 ml). The samples are processed immediately by the Hematology Analyzer from Beckman Coulter model DxH800 series number BB20256.

The study was performed following the General Health Law on Research – Mexico, the Declaration of Helsinki Fortaleza, Brazil and the International Conference on Harmonization Good Clinical Practices. The study was approved by both Ethics Committee (INER/CEI/537/2) and Research Committee (INER/CI/474/20) of INER with the code C91-20.

## Results

### Population characteristics

A total of 191 cases were analyzed, 78% at INER and 22% at ISSEMyM, with a mean age of 48.7 ± 12.7 (median 49; interquartile range [IQR] 40–57 years) and with 75.9% of patients being men. In most cases, one to two comorbidities were associated, with overweight/obesity being the most common in 83.8% of the cases with an average BMI 29.8 ± 5.5 kg/m^2^ (median 29.7; IQR 26.3–32.5 kg/m^2^). At the time of admission, based on the COVID-19 pulmonary severity score by INER, most patients presented with a moderate disease (44.5%), followed by severe disease (43.5%), with a mean percentage of lung involvement of 53.4% ± 28.7 (median 45; IQR 28.5–80). The full and detailed population characteristics per health center can be found in Appendix 1.

Regarding health-center management information, 81 cases (54.5%) fully recovered at INER and 34 cases (81.0%) at ISSEMyM. Confirmatory COVID-19 diagnosis was performed via PCR testing in 169 cases (78.1%), 127 cases at INER and 42 cases at ISSEMyM. For the rest of cases, in which COVID-19 could not be confirmed, these were managed as such based on lung involvement scores and clinical presentation. There were only two cases with low pulmonary involvement of 5%, one with a tobacco index of 3.3 and oxygen saturation of 94% and the second case with a tobacco index of six and an oxygen saturation of 92%, both with a mild clinical presentation.

Table 1 shows the full list of patients' characteristics with their corresponding p-value. Based on the patients' characteristics analyzed, both gender (p = 0.007) and age (p < 0.001) were statistically significant. Specifically for age, the cutoff values of 60 and 70 years presented a significant value with p = 0.038 and 0.042, respectively. It is important to mention that the cutoff point of 60 years is important as it is taken in consideration in the Comorbidity Age Lymphocyte and LDH [CALL] score for predicting outcomes in COVID-19. Regarding patients' comorbidities, different proportions were found by outcome for those who had at least one comorbidity present (p = 0.002). Of the comorbidities analyzed, overweight/obesity (p = 0.006) and hypertension (p = 0.024) were statistically significant.

**Table 1.** General population characteristics grouped in “Recovered” and “Deceased” patients with their respective p-value.

Characteristics		Recovered, n (%)	Deceased, n (%)	Total, n (%)	p-value
Gender	Female	36 (78.3)	10 (21.7)	46 (24.1)	0.007
	Male	79 (54.5)	66 (45.5)	145 (75.9)	
Age	Mean (SD)	46.0 (12.6)	52.7 (11.9)	48.7 (12.7)	<0.001
	Median (IQR)	46 (36–53.5)	52 (45–60)	49 (40–57)	0.001
	≤50	70 (66.0)	36 (34.0)	106 (55.5)	0.091
	>50	45 (52.9)	40 (47.1)	85 (44.5)	
	≤60	102 (63.8)	58 (36.2)	160 (83.8)	0.038
	>60	13 (41.9)	18 (58.1)	31 (16.2)	
	≤70	113 (62.1)	69 (37.9)	182 (95.3)	0.042
	>70	2 (22.2)	7 (77.8)	9 (4.7)	
Comorbidities (d)	Mean (SD)	1.4 (1.0)	1.9 (0.9)	1.6 (1.0)	0.001
	Median (IQR)	1 (1–2.0)	2 (1–2)	2 (1–2)	<0.001
Comorbidities (n)	None	16 (100.0)	0 (0.0)	16 (8.4)	0.002
	1	52 (65.8)	27 (34.2)	79 (41.4)	
	2	32 (48.5)	34 (51.5)	66 (34.6)	
	3	10 (50.0)	10 (50.0)	20 (10.5)	
	4	5 (50.0)	5 (50.0)	10 (5.2)	
Diabetes mellitus	No	84 (61.8)	52 (38.2)	136 (71.2)	0.598
	Yes	31 (56.4)	24 (43.6)	55 (28.8)	
Hypertension	No	96 (64.9)	52 (35.1)	148 (77.5)	0.024
	Yes	19 (44.2)	24 (55.8)	43 (22.5)	
Tobacco exposure	No	88 (63.8)	50 (36.2)	138 (72.3)	0.145
	Yes	27 (50.9)	26 (49.1)	53 (27.7)	
Overweight/obesity	No	26 (83.9)	5 (16.1)	31 (16.2)	0.006
	Yes	89 (55.6)	71 (44.4)	160 (83.8)	
BMI	Mean (SD)	28.5 (4.4)	31.7 (5.9)	29.8 (5.3)	<0.001
	Median (IQR)	28.6 (25.1–30.6)	30.6 (27.6–35)	29.6 (26.3–32.5)	<0.001
Days with symptoms	<5	23 (59.0)	16 (41.0)	39 (20.4)	1
	>5	92 (60.5)	60 (39.5)	152 (79.6)	
Clinical presentation	Mild	22 (100.0)	0 (0.0)	22 (11.5)	<0.001
	Moderate	65 (76.5)	20 (23.5)	85 (44.5)	
	Severe	27 (32.5)	56 (67.5)	83 (43.5)	
	No data	1 (100.0)	0 (0.0)	1 (0.5)	
O_2_ saturation (%)	Mean (SD)	82.1 (12.5)	67.9 (15.3)	76.5 (15.3)	<0.001
	Median (IQR)	86 (80.5–89)	70 (56.8–81.2)	83 (69–87)	<0.001
	≥85	69 (81.2)	16 (18.8)	85 (44.5)	<0.001
	<85	45 (42.9)	60 (57.1)	105 (55.0)	
	No data	1 (100.0)	0 (0.0)	1 (0.5)	
Health center	INER	81 (54.4)	68 (45.6)	149 (78.0)	0.003
	ISSEMyM	34 (81.0)	8 (19.0)	42 (22.0)	

d: Distributional data; INER: Instituto Nacional de Enfermedades Respiratorias; IQR: Interquartile range; ISSEMyM: Instituto de Seguridad Social del Estado de México y Municipios; n: Counting data; SD: Standard deviation.

**Figure 1.**
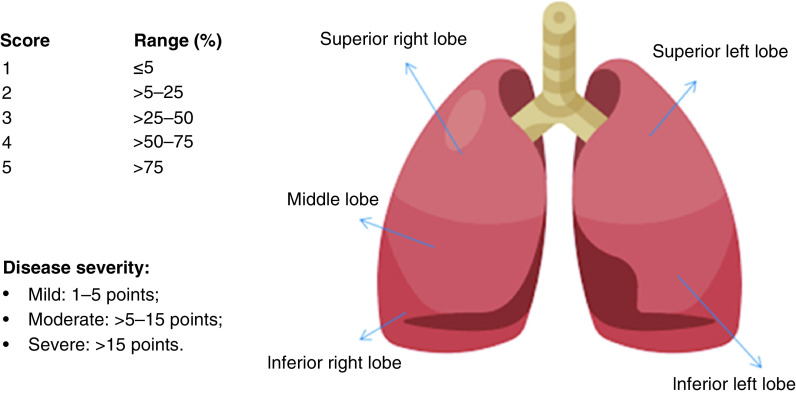
COVID-19 pulmonary involvement via computed tomography scan. System proposed by the INER. This system proposed by the Radiology Department evaluates the pulmonary damage caused by COVlD-19 via CT-scan. The system is a semi-quantitative assessment of lung involvement per lobe and has been used at a national scale since the beginning of the pandemic. INER: Instituto Nacional de Enfermedades Respiratorias.

With the goal of evaluating COVID-19-associated pulmonary damage and classifying disease severity, the INER created a computed tomography-based scale used at a national level when managing a patient with COVID-19, which can be found in Figure 1. Taking into consideration the clinical evaluation at time of admission and the healthcare center where they were treated, zero patients classified as having mild disease passed away; however, one-fifth and two-thirds of patients with moderate and severe disease passed away, respectively. When comparing health centers, there was a higher number of deaths in the patients treated at INER compared with those treated at ISSEMyM. These differences are due to the high number of cases treated at INER and the severity of disease that each health center treated. Similar numbers of cases with mild and moderate disease were treated in both INER and ISSEMyM, with a total of 3.6% of patients with mild disease (10.7% INER vs 14.3% ISSEMyM) and 15.8% with moderate disease (41.4% INER vs 57.1% ISSEMyM). However, a greater difference was found for severe disease, with a total of 21.8% classified as severe, 48.0% INER versus 26.2% ISSEMyM.

### Clinical & biochemical markers

When evaluating the percentage of COVID-19 pneumonia, biochemical markers and hematological and immune cell values, the authors observed that multiple markers were statistically significant in both mean values and median values. Regarding the percentage of lung involvement, this variable was statistically significant (p < 0.001) and when comparing the percentage between deceased patients versus recovered, deceased patients having a two-times higher median value. When considering the 50% and 60% cutoff values for pneumonia percentage, these cutoff points also indicated an increased risk of death (p < 0.001).

The main objective of the study was to evaluate the impact that each biochemical and serum marker had on patient mortality, with the goal of identifying values that should be taken in consideration when managing a patient with COVID-19. The biochemical markers that were statistically significant were albumin (p < 0.001), lactate dehydrogenase (LDH) (p < 0.001), ferritin (p = 0.021), D-dimer (p < 0.001), procalcitonin (p < 0.001), pro-brain natriuretic peptide (p = 0.043), leukocyte count (p < 0.001), neutrophil count (p < 0.001), lymphocyte count (p < 0.001) and eosinophil count (p < 0.0001).

As previously mentioned, one of the most important predictive factors being studied in COVID-19 are the ratios that exist between different immunological cell values, and in this study the authors demonstrate that the MLR, NLR, PLR, LLR and NI were all statistically significant with a p-value <0.001. When evaluating each ratio, the authors observed that every ratio was significantly higher in the deceased group compared with recovered patients.

#### Leukocyte-to-lymphocyte ratio

Regarding the LLR, there was a two-times increase in deceased patients compared with recovered patients (p < 0.001), with a mean value of 10.2 versus 19.8 and a median of 7.4 versus 14.3, respectively. With the goal of making clinical decision easier, a cutoff point of 7.7 was established and appeared to be statistically significant. These results make the LLR an independent predictive prognostic factor associated with an increased mortality, especially with a value >7.7.

#### Neutrophil-to-lymphocyte ratio

This ratio appeared to be two-times higher in deceased patients compared with recovered patients, with a mean value of 17.7 versus 9.1 and a median of 13.5 versus 6.2, respectively. A cutoff point was also established, being ten. This cutoff point was also statistically significant when comparing deceased patients to patients who recovered. This makes the NLR a mortality prognostic value that can be used when evaluating patients with COVID-19, especially when the ratio is >10.

#### Monocyte-to-lymphocyte ratio

The MLR also demonstrated significant differences between the group of deceased patients versus recovered, with a mean value of 759.3 versus 539.5 and a median value of 666.7 versus 452.3, respectively. Additionally, a cutoff point to facilitate clinical decisions was established with a value of 500. This cutoff point was statistically significant (p = 0.002), indicating that an MLR >500 is associated with an increased risk of mortality in patients diagnosed with COVID-19.

#### Platelet-to-lymphocyte ratio

Regarding the PLR, differences were also found between the deceased patients versus recovered, with a mean value of 369.1 versus 272.6 and a median of 306.7 versus 225, respectively. As performed with the previously mentioned ratios, a cutoff point was established, being in this case 250. This cutoff point was statistically significant (p < 0.001), leading to the conclusion that the PLR can be used as a mortality prognostic biomarker, especially when the value is >250.

#### Nutritional index

The last biomarker taken in consideration was the NI. This NI also appeared to be significantly different between deceased patients and recovered patients, with a mean value of 326.7 versus 359.7 and a median of 333 versus 363, respectively. The cutoff point established was 362, which appeared to be statistically significant (p = 0.004). This indicates that an NI value <362 is associated with an increased risk of mortality in patients diagnosed with COVID-19.

The full list of clinical, biochemical markers and their respective ratios evaluated for both groups of patients (deceased vs recovered) with their respective p-value can be found detailed in Table 2.

**Table 2.** Pneumonia percentage and biochemical and hematological population characteristics, grouped in ‘Recovered’ and ‘Deceased’ patients with their respective p-value.

Characteristics		Recovered, n (%)	Deceased, n (%)	Total, n (%)	p-value
Pneumonia (%)	Mean (SD)	42.5 (26.4)	69.9 (23.8)	53.4 (28.7)	<0.001
	Median (IQR)	35 (25–59.0)	80 (53.2–85)	45 (28.5–80)	<0.001
	≤50	80 (80.8)	19 (19.2)	99 (51.8)	<0.001
	>50	34 (37.4)	57 (62.6)	91 (47.6)	
	No data	1 (100.0)	0 (0.0)	1 (0.5)	
	≤60	88 (81.5)	20 (18.5)	108 (56.5)	<0.001
	>60	26 (31.7)	56 (68.3)	82 (42.9)	
	No data	1 (100.0)	0 (0.0)	1 (0.5)	
Albumin (g/dl)	Mean (SD)	3.5 (0.5)	3.2 (0.6)	3.4 (0.6)	<0.001
	Median (IQR)	3.5 (3.2–3.8)	3.3 (3–3.5)	3.4 (3.1–3.8)	<0.001
	≤3.4	45 (48.9)	47 (51.1)	92 (48.3)	0.004
	>3.4	69 (70.4)	29 (29.6)	98 (51.3)	
	No data	1 (100.0)	0 (0.0)	1 (0.5)	
Hemoglobin (g/dl)	Mean (SD)	14.9 (2.1)	15.1 (2.1)	14.9 (2.1)	0.483
	Median (IQR)	15.3 (14–16.2)	15.3 (14.1–16.4)	15.3 (14–16.3)	0.428
Platelets (10^3^/mm^3^)	Mean (SD)	236.5 (115.0)	220.3 (91.2)	230.0 (106.2)	0.281
	Median (IQR)	206 (162–270.5)	199.5 (156.8–290.8)	203.5 (157–286.2)	0.579
LDH (units/l)	Mean (SD)	433.5 (205.4)	645.6 (341.6)	517.9 (286.7)	<0.001
	Median (IQR)	392.0 (282.5–529.6)	573.9 (423.9–726.2)	464.0 (324.9–622.5)	<0.001
	<250	19 (86.4)	3 (13.6)	22 (11.5)	0.015
	≥250	96 (56.8)	73 (43.2)	169 (88.5)	
Ferritin (ng/ml)	Mean (SD)	1550.1 (4051.0)	1906.6 (1740.3)	1674.1 (3420.6)	0.558
	Median (IQR)	1003.4 (491.4–1429.2)	1290.8 (848.5–2249.5)	1054.0 (603.0–1572.6)	0.021
	≤1000	30 (71.4)	12 (28.6)	42 (22.0)	0.354
	>1000	30 (60.0)	20 (40.0)	50 (26.2)	
	No data	55 (55.6)	44 (44.4)	99 (51.8)	
D-dimer (μg/ml)	Mean (SD)	6.5 (56.4)	4.3 (7.8)	5.6 (43.9)	0.686
	Median (IQR)	0.6 (0.4–1.0)	1.4 (0.8–3.5)	0.8 (0.5–1.8)	<0.001
	≤500	112 (59.9)	75 (40.1)	187 (97.9)	1
	>500	1 (100.0)	0 (0.0)	1 (0.5)	
	No data	2 (66.7)	1 (33.3)	3 (1.6)	
Procalcitonin (ng/ml)	Mean (SD)	1.8 (9.6)	1.8 (3.6)	1.8 (7.8)	0.957
	Median (IQR)	0.1 (0.1–0.6)	0.4 (0.1–1.4)	0.2 (0.1–0.8)	<0.001
	≤0.2	69 (72.6)	26 (27.4)	95 (49.7)	0.001
	>0.2	46 (47.9)	50 (52.1)	96 (50.3)	
pro-BNP (pg/ml)	Mean (SD)	64.7 (107.7)	150.8 (210.2)	113.2 (177.0)	0.054
	Median (IQR)	25.8 (10–56.8)	48.9 (25.5–212.4)	33.9 (11.6–108.8)	0.043
	≤40	15 (50.0)	15 (50.0)	30 (15.7)	0.442
	>40	9 (36.0)	16 (64.0)	25 (13.1)	
	No data	91 (66.9)	45 (33.1)	136 (71.2)	
	≤30	15 (62.5)	9 (37.5)	24 (12.6)	0.027
	>30	9 (29.0)	22 (71.0)	31 (16.2)	
	No data	91 (66.9)	45 (33.1)	136 (71.2)	
Leukocytes (10^3^/mm^3^)	Mean (SD)	8.4 (3.9)	11.0 (5.4)	9.4 (4.7)	<0.001
	Median (IQR)	7.3 (5.6–10.2)	10.1 (7.0–13.6)	8.1 (6.0–11.9)	<0.001
	≤4	7 (70.0)	3 (30.0)	10 (5.2)	0.751
	>4	108 (59.7)	73 (40.3)	181 (94.8)	
	≤10	85 (69.7)	37 (30.3)	122 (63.9)	0.001
	>10	30 (43.5)	39 (56.5)	69 (36.1)	
Neutrophils (10^3^/mm^3^)	Mean (SD)	7.2 (3.9)	9.7 (4.7)	8.2 (4.4)	<0.001
	Median (IQR)	6.3 (4.5–9.4)	9.4 (6.1–11.8)	7.7 (5–10.4)	<0.001
	≤10	94 (67.6)	45 (32.4)	139 (72.8)	0.001
	>10	21 (40.4)	31 (59.6)	52 (27.2)	
Lymphocytes (10^3^/mm^3^)	Mean (SD)	1.1 (0.5)	0.8 (0.5)	1.0 (0.5)	<0.001
	Median (IQR)	1.0 (0.7–1.4)	0.7 (0.5–0.9)	0.8 (0.6–1.2)	<0.001
	≤1	59 (47.2)	66 (52.8)	125 (65.4)	<0.001
	>1	56 (84.8)	10 (15.2)	66 (34.6)	
Monocytes (10^3^/mm^3^)	Mean (SD)	490.2 (243.8)	486.1 (240.2)	488.5 (241.7)	0.91
	Median (IQR)	450 (300–600)	400 (300–600)	400 (300–600)	0.756
MLR	Mean (SD)	539.5 (349.7)	759.3 (438.1)	626.9 (400.9)	<0.001
	Median (IQR)	452.3 (328.1–666.7)	666.7 (418.1–1000)	500 (375–800)	<0.001
	≤500	69 (71.1)	28 (28.9)	97 (50.8)	0.002
	>500	43 (48.3)	46 (51.7)	89 (46.6)	
	No data	3 (60)	2 (40)	5 (2.6)	
NLR	Mean (SD)	9.1 (8.3)	17.7 (16.7)	12.6 (13.0)	<0.001
	Median (IQR)	6.2 (3.8–10.6)	13.5 (7.6 a 20.9)	8.4 (5.1 a 16.2)	<0.001
	≤10	83 (74.8)	28 (25.2)	111 (58.1)	<0.001
	>10	32 (40)	48 (60)	80 (41.9)	
PLR	Mean (SD)	272.6 (192.6)	369.1 (240.4)	311.2 (217.6)	0.004
	Median (IQR)	225 (151.5–315.9)	306.7 (213.2–454.2)	257.6 (165.6–384.7)	<0.001
	<250	67 (73.6)	24 (26.4)	91 (47.6)	<0.001
	≥250	47 (48.0)	51 (52.0)	98 (48.2)	
	No data	1 (50.0)	1 (50.0)	1 (0.5)	
LLR	Mean (SD)	10.2 (8.4)	19.8 (19.1)	14.1 (14.4)	<0.001
	Median (IQR	7.4 (4.8–12.5)	14.3 (8.9–22.8)	9.7 (5.8–16.4)	<0.001
	<7.7288	64 (82.1)	14 (17.9)	78 (40.8)	<0.001
	>7.7288	51 (45.1)	62 (54.86)	62 (59.2)	
Derived NLR	Mean (SD)	1.1 (0.4)	1.1 (0.7)	1.1 (0.5)	0.584
	Median (IQR)	0.9 (0.9–1.0)	0.9 (0.9–1.0)	0.9 (0.9–1.0)	0.246
NI	Mean (SD)	359.7 (51.7)	326.7 (56.7)	346.5 (56.0)	<0.001
	Median (IQR)	363.5 (326.6–390.4)	333 (301–356.8)	347.3 (318–381.5)	<0.001
	≥362	57 (77.0)	17 (23.0)	74 (38.7)	0.004
	<362	56 (48.5)	59 (51.3)	115 (60.2)	<0.001
	No data	2 (100.0)	0 (0.0)	2 (1.0)	

IQR: Interquartile range; LDH: Lactate dehydrogenase; LLR: Leukocytes-to-lymphocytes ratio; MLR: Monocytes-to-lymphocytes ratio; NI: Nutritional index; NLR: Neutrophils-to-lymphocytes ratio; PLR: Platelets-to-lymphocytes ratio; pro-BNP: Pro-brain natriuretic peptide; SD: Standard deviation.

### Adjusted odds ratio analysis (univariable & multivariable)

With the goal of evaluating if the effect of some variables was attenuated when using a univariate or multivariate odds ratio (OR), the authors explored the specific patients' conditions per group, in which cutoff points with significant value were included in both deceased and recovered patients. In case one of the variables was analyzed with more than one cutoff point, the most significant value was taken into consideration and was considered as ‘greater protection’ or ‘greater risk’. Variables in which likelihood ratios were not able to be estimated were excluded either because there were no health center reference data levels or because the data was low to be reliable. Based on these analyses, comorbidities and clinical presentation at time of diagnosis were excluded. The intervals were calculated using the Wald estimate and the results of these analyses can be found in Appendices 2–4. Each table corresponds to a different model of analysis performed with the goal of identifying the significant variables that correspond as prognostic mortality factors.

**Figure 2.**
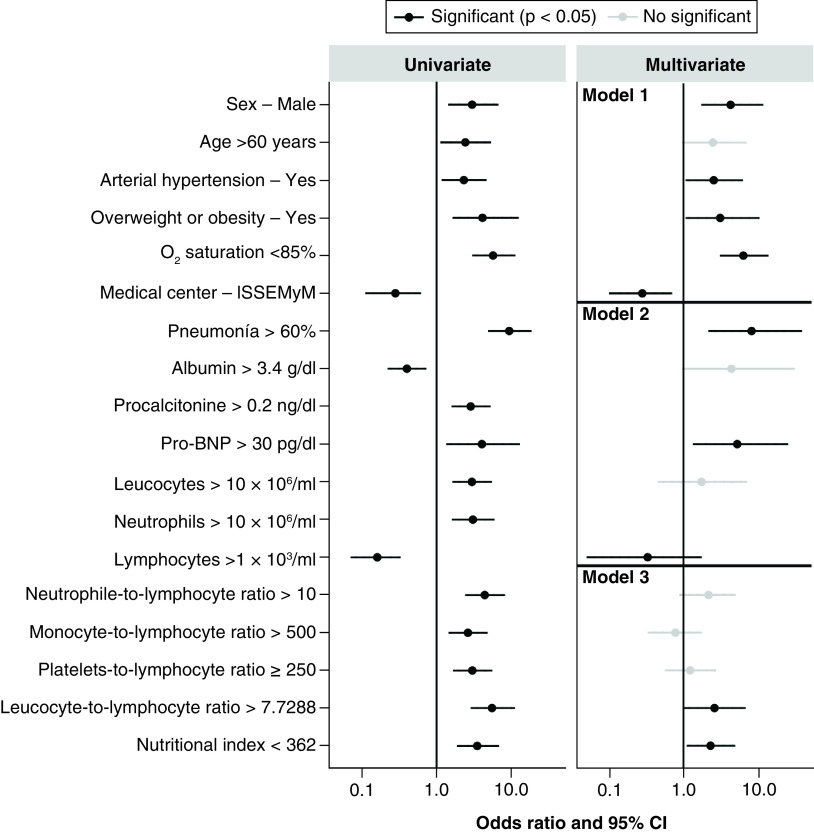
Odds ratio per prognostic factor variable.

Based on these statistical models, the authors can observe that model 3 demonstrated in Table 3 is most significant and suits the data in this study. Results with their significance and OR can be found in Table 3 & Figure 2. Based on this model, all the ratios analyzed (NLR, MLR, PLR, LLR and NI) were statistically significant in the univariate analysis, and in the multivariate analysis both the LLR and NI were statistically significant with p-values of 0.049 and 0.028, respectively. This model allows us to identify those biochemical values that are significant and useful as mortality prognostic factors, which corresponds to an LLR >7.7 and NI <362. The rest of the clinical characteristics and hematological and biochemical values significant in the univariate analysis should not be completely excluded in future analysis, as they are still associated as mortality prognostic factors. The authors believe these results were not fully significant due to a limited number of patients and probably due to specific population characteristics that may differ from previously published studies.

**Table 3.** Model 3 – adjustment based on biochemical and hematological parameters.

Variable	Cutoff point	Recovered	Deceased	OR (univariable)	OR (multivariable)
NLR	≤10	83 (74.8)	28 (25.2)	–	–
	>10	32 (40.0)	48 (60.0)	4.45 (2.42–8.36, p < 0.001)	2.17 (0.94–5.06, p = 0.069)
MLR	≤500	69 (71.1)	28 (28.9)	–	–
	>500	43 (48.3)	46 (51.7)	2.64 (1.45–4.87, p = 0.002)	0.78 (0.33–1.77, p = 0.597)
PLR	<250	67 (73.6)	24 (26.4)	-	-
	≥250	47 (48.0)	51 (52.0)	3.03 (1.66–5.65, p < 0.001)	1.23 (0.56–2.66, p = 0.596)
LLR	≤7.7288	64 (82.1)	14 (17.9)	–	–
	>7.7288	51 (45.1)	62 (54.9)	5.57 (2.86–11.38, p < 0.001)	2.61 (1.01–6.84, p = 0.049)
NI	≥362	57 (77.0)	17 (23.0)	–	–
	<362	56 (48.7)	59 (51.3)	3.53 (1.87–6.94, p < 0.001)	2.31 (1.10–4.97, p = 0.028)

Variables included: NLR, MLR, PLR, LLR, NI.

Model metrics:

Total n = 191; n at the model = 182; Missing data = ten.

AIC = 221.7; C-statistic = 0.755; H&L test: χ2(8) = 10.35; p = 0.241.

AIC: Akaike information criterion; H&L: Hosmer–Lemeshow test; LLR: Leukocytes-to-lymphocytes ratio; MLR: Monocytes-to-lymphocytes ratio; NI: Nutritional index; NLR: Neutrophils-to-lymphocytes ratio; OR: Odds ratio; PLR: Platelets-to-lymphocytes ratio.

## Discussion

COVID-19 infects alveolar epithelial cells via angiotensin-converting enzyme-2 receptors releasing inflammatory factors and activating macrophages in the alveolar tissue, leading to a massive inflammatory cascade that damages multiple organs, but mainly the lung parenchyma. This massive inflammation may cause severe lung injury and acute respiratory distress syndrome leading to an increased mortality. Severe cases of COVID-19 often present in the elderly or patients with chronic diseases and comorbidities such as systemic arterial hypertension, obesity and diabetes. One of the most studied comorbidities is obesity, which by itself is considered a proinflammatory state and therefore is associated with an increased mortality in COVID-19 patients [21,22]. The Mexican population is at high risk of developing severe COVID-19 due to the high prevalence of overweight, with Mexico ranked as the country with the second-highest prevalence of obesity and with 70% of the population being overweight [23,24]. It is important to mention that obesity by itself is associated with an increased morbidity and mortality, and when associated with COVID-19 multiple meta-analyses have proven that the evolution to a severe disease is increased in those who are in the overweight/obesity category and even more in those who carry additional comorbidities such as diabetes, hypertension, dyslipidemia and cardiovascular diseases [25].

Regarding the use of blood tests measuring hematological and immune cells, studies have associated a decrease in the lymphocyte count and an increase in the neutrophil count with severe cases of COVID-19, such as the current study demonstrated. Additionally, patients who develop severe infection their oxygen saturation dropped lower than 88% in more than 30% of cases and this is associated with an increased risk of hospitalization [26]. This study considered an oxygen saturation cutoff point of 85% at admission to evaluate severity, demonstrating this saturation level or lower to be indicative of a higher mortality and was considered a mortality prognostic value both independently (OR 5.75; CI 95%: 3.01–11.29; p < 0.001) and in combination with other parameters (OR 6.37; CI 95%: 2.09–13.89; p < 0.001).

When analyzing the results of pulmonary damage percentage, cellular count and biochemical markers, these behaved as independent mortality prognostic factors similar to what has been demonstrated in previous studies [27]. An interesting finding in this study was regarding albumin levels, which demonstrated an OR interval inversion from the univariate (OR 0.40; CI 95%: 0.22–0.73; p = 0.001) to the multivariate analysis (OR 4.31; CI 95%: 0.90–31.35; p = 0.095). NLR has been identified in a study by Zahorec *et al.* as an early marker for severe COVID-19 disease progression [28]. Based on these results, multiple ratios have been studied, such as the NLR, which was elevated in deceased patients; however, different cutoff points have been established. In Mexico, a country with high prevalence of severe COVID-19, multiple authors have described different cutoff points as mortality prognostic factors. Del Carpio Orantes mentioned a cutoff point of 20.4 ± 16.9 in the deceased patients versus 7.5 ± 4.9 in recovered patients, while Albarrán-Sánchez *et al.* had a cutoff point of 17.66 versus 8.31, respectively [29,30]. Albarrán *et al.* also demonstrated that an NLR >12 predicts mortality, similar to the results from this study, which established a cutoff value >10 with an OR of 4.45 (CI 95%: 2.42–8.36; p < 0.001). These results demonstrate that NLR is associated with disease severity and can be used as a mortality prognostic factor.

Similar studies to the ones mentioned above did not evaluate albumin levels. Even though albumin is an acute phase reactant that is expected to change in inflammatory processes, there is limited evidence regarding COVID: one study demonstrated that the albumin/C-reactive protein ratio can be a useful prognostic indicator of disease severity, but it does not predict mortality [31]. This study did evaluate albumin levels, identifying them as a mortality prognostic factor, and evaluated albumin in the NI. The NI in this study was considered an independent mortality predictive factor in patients with COVID-19. Additionally, a cutoff point <362 at the time of admission was associated with a twofold increase in hospitalized patient mortality. This cutoff value had a 62.63% precision, with an area under the curve receiver operating characteristic of 67.62%, 80.26% sensitivity and 50.88% specificity. The authors only identified one previous study, by Xue *et al.*, that also evaluated the NI and established a cutoff point of 340.5 obtaining a precision of 73.68%, 86.21% sensitivity and 60.71% specificity [32]. These results can be compared and are similar to the population of the current study, making the NI an important mortality prognostic factor that must be taken in consideration in the management of COVID-19 patients in both the Chinese and the Latino/Mexican population.

The LLR was also evaluated in this study and demonstrated a significant value with a p < 0.001. A cutoff value of 7.7 was established based on this study's population data: this cut-off had an 81.58% sensitivity, 55.65% specificity with a 65.97% precision and an area under the curve receiver operating characteristic of 73.74%. Additionally, the LLR 7.7 cutoff value demonstrated an OR of 5.57 in the univariate analysis (CI 95%: 2.86–11.38; p < 0.001) and an OR of 2.61 in the multivariate analysis (CI 95%: 1.01–6.84; p = 0.049). There results make the LLR an important mortality prognostic factor that must be taken in consideration when evaluating a COVID-19 patient, using the 7.7 cutoff point for clinical decisions.

Based on these results and comparing them with previous studies, healthcare professionals treating COVID-19 patients must use these hematological ratios, specially the LLR and NI, with the goal of identifying those with an increased risk of developing severe disease and mortality. This study proposes the cutoff points of 7.7 for the LLR and 362 for the NI, to be used as mortality prognostic factors to prioritize treatment and make fast medical decisions using evidence-based medicine in a current pandemic situation.

## Conclusion

Both an LLR >7.7 and an NI <362 are mortality predictor factors that must be used in the management of COVID-19 hospitalized patients. Additional prognostic factors of mortality and severity that must be taken in consideration include an oxygen saturation <85%, an NLR >10 and diminished albumin levels. We encourage further analysis on ratios such as the MLR and PLR associating them with an increased mortality. Our study confirms that these ratios can be used in the management of COVID-19 Latino patients, an under-represented population in many studies and of extreme importance.

## Limitations

One main limitation of the present study was the limited number of patients, which was probably associated with not observing a significant value in the rest of the variables analyzed. Other limitations include being a retrospective transversal study and not having a unified treatment management.

## Future perspective

Serum inflammatory biomarkers help in making fast and critical medical decisions, especially in an ongoing pandemic. Identifying prognostic disease severity and mortality biomarkers in a severe disease such as COVID-19 will help in future pandemics by allowing health centers to make adequate treatment decisions, prioritize management and use health resources adequately. Additionally, these inflammatory biomarkers are most likely associated prognostic factors in important diseases such as neoplasias, autoimmune diseases and infectious diseases, which will help guide treatment.

**Summary points**

- The most significant serological haemato-immunological inflammatory biomarker ratios identified as mortality prognostic factors in the Latino population include a leukocyte-to-lymphocyte ratio >7.7 and a nutritional index <362, and these factors must be used in the management of COVID-19 patients.
- Additional serological ratios associated with an increased mortality and disease severity include a neutrophil-to-lymphocyte ratio >10, monocyte-to-lymphocyte ratio >500 and platelet-to-lymphocyte ratio >250.
- Prognostic factors that must be taken in consideration independently include oxygen saturation <85% and diminished albumin levels.
- Albumin levels demonstrate an odds ratio (OR) interval inversion from the univariate (OR 0.40; CI 95%: 0.22–0.73; p = 0.001) to the multivariate analysis (OR 4.31; CI 95%: 0.90–31.35; p = 0.095).
- Age cutoff values of 60 and 70 years are associated with an increased mortality and disease severity.
- A 50% and 60% cutoff value for pneumonia percentage damage indicates an increased risk of death.
